# Mixture of Peanut Skin Extract and Fish Oil Improves Memory in Mice via Modulation of Anti-Oxidative Stress and Regulation of BDNF/ERK/CREB Signaling Pathways

**DOI:** 10.3390/nu8050256

**Published:** 2016-04-28

**Authors:** Lan Xiang, Xue-Li Cao, Tian-Yan Xing, Daisuke Mori, Rui-Qi Tang, Jing Li, Li-Juan Gao, Jian-Hua Qi

**Affiliations:** 1 College of Pharmaceutical Sciences, Zhejiang University, Hangzhou 310058, China; 11319006@zju.edu.cn (X.-L.C.); ricky.tang@163.com (R.-Q.T.); 11419008@zju.edu.cn (J.L.); gaolijuan04141002@126.com (L.-J.G.); 2 Hangzhou Napochi Pharmaceutical Co. Ltd., Hangzhou 310018, China; hznqrsw@163.com; 3 Gifu Shellac Mfg. Co., Ltd., 1-27, Kanonishimaru-cho, Gifu 500, Japan; numbermidori@gmail.com

**Keywords:** brain-derived neurotrophic factor, fish oil, ERK, oxidative stress, peanut skin extract

## Abstract

Long-term use of fish oil (FO) is known to induce oxidative stress and increase the risk of Alzheimer’s disease in humans. In the present study, peanut skin extract (PSE), which has strong antioxidant capacity, was mixed with FO to reduce its side effects while maintaining its beneficial properties. Twelve-week Institute of Cancer Research (ICR) mice were used to conduct animal behavior tests in order to evaluate the memory-enhancing ability of the mixture of peanut skin extract and fish oil (MPF). MPF significantly increased alternations in the Y-maze and cognitive index in the novel object recognition test. MPF also improved performance in the water maze test. We further sought to understand the mechanisms underlying these effects. A significant decrease in superoxide dismutase (SOD) activity and an increase in malonyldialdehyde (MDA) in plasma were observed in the FO group. The MPF group showed reduced MDA level and increased SOD activity in the plasma, cortex and hippocampus. Furthermore, the gene expression levels of brain-derived neurotrophic factor (BDNF) and cAMP responsive element-binding protein (CREB) in the hippocampus were increased in the MPF group, while phosphorylation of protein kinase B (AKT), extracellular signal-regulated kinase (ERK) and CREB in the hippocampus were enhanced. MPF improves memory in mice via modulation of anti-oxidative stress and activation of BDNF/ERK/CREB signaling pathways.

## 1. Introduction

Fish oil (FO) is a widely used nutrition supplement. It is rich in omega-3 fatty acids, specifically docosahexaenoic acid (DHA) and eicosapentaenoic acid (EPA). Previous studies have indicated that FO can reduce blood fat [1] and prevent Alzheimer’s disease (AD) [2]. FO also exhibits anti-inflammatory [3], anti-cancer [4], and anti-aging [5] effects. However, negative reports concerning FO consumption have emerged in recent years. The oxidation products of FO are harmful to mitochondria functions [6,7]. Furthermore, FO replaces critical omega-6 metabolites, thus modifying tissue structure, and reducing prostacyclin production, which increases the risk of cardiovascular diseases [8]. Furthermore, prolonged or excessive consumption of FO containing the oxidized product polyunsaturated fatty acid oxide leads to production of free radicals and induction of cellular senescence, which involves senile plaque formation and tissue damage. These problems limit the use of FO as a nutrient supplement. Therefore, reassessment of the function and reliability of FO is imperative.

Oxidative stress, insulin resistance and inflammation interrelate. Oxidative stress can lead to insulin resistance and inflammation. Conversely, both insulin resistance and inflammation can also increase oxidative stress. Several studies have shown that all of these factors can be induced by a high fat diet and can impair cognitive performance and memory decline [9,10,11]. Therefore, further studies are necessary for improvement of cognitive function of the aging brain by using anti-oxidative, anti-diabetic and anti-inflammatory agents.

Peanut skin is a protective pink-red layer with astringent taste. It is rich in phenolics and other health-promoting compounds. Peanut skin extract (PSE) mainly contains 3-procyanidins, 4-anthocyanins, 2-flavanols, and 5-flavonols. Resveratrol also exists in PSE and its concentration is higher than that in peanut kernels [12]. The anti-inflammatory, anti-cardiovascular disease, anti-cancer, anti-obesity and anti-diabetic properties of many polyphenolic compounds have been investigated recently [13,14,15,16,17]. Peanut skin polyphenols, particularly procyanidine, improve lipid homeostasis, reduce inflammation, and act as natural antioxidants and antimicrobial agents [18,19,20]. In the current study, we used PSE to alleviate the side effects of FO and sought to determine whether MPF improves memory in mice via its anti-oxidative effects or regulation of gene expression.

## 2. Materials and Methods

### 2.1. PSE, FO and MPF

PSE and FO with 20% DHA and 8% EPA were obtained from Gifu Shellac Mfg. Co., Ltd., Gifu, Japan. MPF was composed of PSE and FO at a 1:2 ratio by mass. The vehicle contained 40% glycerin, 6% emulsifier, and 54% water. Samples were prepared by dissolving PSE, FO, and MPF in the vehicle to get final concentrations of 15% PSE, 30% FO, and 15% PSE + 30% FO, respectively. The effective dose of fish oil for use as supplements has been shown to be 20–40 mg/kg for adults. Therefore, 20 mg/kg of FO was used in our study.

### 2.2. Neurite Outgrowth Assay

Bioassay was performed as described in a previous study [21]. Firstly, 2 × 10^4^ rat pheochromocytoma (PC12) cells were placed in each well of a 24-well microplate and cultured in 5% CO_2_ incubator at 37 °C for 24 h. After that, 1 mL of serum-free dulbecco’s modified eagle medium that contain test samples (PSE at doses of 0.3, 1 and 3 µg/mL; FO at doses of 0.3, 1 and 3 µg/mL; MPF at doses of 0.3 + 0.3, 0.3 + 1, 0.3 + 3, 1 + 0.3, 1 + 1, 1 + 3 µg/mL) and dimethyl sulfoxide (DMSO) (0.5%) was used to replace old medium and incubated for two days. Nerve growth factor (NGF) was used as positive control. Cells were observed using a phase-contrast microscope (Olympus, Model CKX41, Tokyo, Japan) every 24 h. Approximately 100 cells were counted from a random region of the culture dish. If the outgrowth of a PC12 cell was longer than the diameter of the cell body, the cell was determined to be neurite-bearing. Independent experiments were repeated thrice and the results are expressed as mean ± SEM.

### 2.3. Animal Study and Experimental Design

Twelve-week-old male ICR mice (*n* = 60) were used as experimental animals (Zhejiang Academy of Medical Sciences, Hangzhou, China). The mice were fed in a clean room at 23 ± 1 °C with a 12:12 light-dark cycle and fed with a commercial diet (Zhejiang Academy of Medical Sciences, Hangzhou, China) *ad libitum*. All experiments were performed according to the Guide by the Animal Ethics Committee of Medical School, Zhejiang University (Permit Number: ZJU201401101005). The mice were divided into six groups. The control group received vehicle treatment. The PSE group received PSE at 10 mg/kg body weight per day. The FO group received FO at 20 mg/kg body weight per day. Three MPF groups received MPF at 0.03, 3 and 30 mg/kg body weight per day with oral administration. The animals were administered with samples for 5 weeks and then subjected to the Y-maze and novel objects recognition (NOR) test. The water maze experiment was performed on the sixth week. After finishing animal behavior experiments, blood was collected from mice orbit with capillary and the treated mice were killed with neck dislocation. Brain and plasma samples of the mice were taken quickly and then frozen at −20 °C.

#### 2.3.1. Y-Maze Memory Test

The mice were tested for spontaneous alternations using the Y-maze as previously reported [22]. The Y-maze used in the present study has three equal arms with 120° angle. The mice were placed in the “start” arm of the Y-maze and left to roam freely for 8 min. Three continuous choices in three different arms were considered to be alternations. The average of percentage alternations was plotted.

#### 2.3.2. NOR Memory Tasks

After completion of the Y-maze test, the NOR test was performed. The novel object apparatus consisted of a white plastic box (46 × 26 × 20 cm), two identical objects (2 × 12 cm plastic flashlights) and a novel object (5 × 11 cm coffee can). At first, each mouse was placed in the empty open field of the box for 5 min for habituation on Day 1. The area was cleaned with 75% ethanol solution well ahead of time to ensure that no olfactory cues were present. The next day, the test of the NOR task was performed, which consisted of a training trial and a retention phase. In the training trial, the mice were exposed to the same arena where two identical objects were placed in opposite sides at equal distances for 5 min. The time spent exploring the two objects by each mouse were measured. After 1 h of training trial, one of two identical objects was replaced with a novel object. The animals were removed from the arena and exposed for 5 min again. The time spent exploring each object was recorded. Exploration was fixed as the mice used the nose or forepaws to sniff or touch the objects. A discrimination index (DI) was represented with the percentage of exploring time on the novel object divided by the total time spent exploring both objects.

#### 2.3.3. Morris Water Maze Test

The Morris water maze test was performed after sample administration for 5 weeks. The apparatus comprised of a movable platform (14 cm diameter) and a circular tank (1.2 m diameter, 50 cm depth). In addition, a digital camera connected to a computer hung above the tank and was used to track mice movement. Before the experiment, water was added up to 34 cm depth and warmed to 22 ± 1 °C. Black ink was added to make the water opaque and to hide the platform. During training, the tank was divided into four quadrants. Each mouse underwent four trials in four quadrants in 1 day. The mice learned to run away from the water by finding the platform under the water in the center of quadrant 1 in the tank. The experiment was performed in 4 days. The mice which could not locate the platform within 120 s were put on the platform and allowed to stay there for 10 s. The probe trials were done on the fifth day after removing the platform. In every trial, the mouse was placed in the water back to the front from one of four starting points. The time to find the platform in training trials and times of crossing platform in the training trial and probe trial were measured by reviewing the video recordings. Data from four tests conducted each day were averaged for statistical analysis.

### 2.4. SOD Activity and MDA Level in the Plasma, the Cerebral Cortex and the Hippocampus

Plasma samples were obtained by centrifuging the blood at 10,000 rpm for 10 min at normal temperature and depot at −20 °C until analysis. Approximately 50 mg of cerebral cortex samples or one hippocampus were homogenized in cold phosphate-buffered saline at a 1:9 volume ratio, sonicated thrice for 1 min each, and then centrifuged at 12,000 rpm for 15 min at 4 °C. The supernatants were used for analysis. The samples were assayed for SOD activity and MDA using commercially available T-SOD and MDA assay kits (Bioengineering Institute of Nanjing Jiancheng Company, Nanjing, China) according to the manufacturer’s protocols. We selected 5.5 µL of basic plasma solution and 25 µL of 1% cerebral cortex or hippocampus homogenate of each sample as optimal quantities to measure the total SOD activity. Simultaneously, approximately 20 µL of plasma, 20 µL of 10% cerebral cortex and 40 µL of 10% hippocampus homogenate were used to measure MDA. The protein concentrations of the cerebral cortex samples were spectrophotometrically measured using a Bio-Rad protein assay kit (Bio-Rad Laboratories, Hercules, CA, USA) at 595 nm.

### 2.5. Real-Time PCR Analysis

Approximately 50 mg of the cerebral cortex and hippocampus samples were obtained. Extraction of total RNA, reverse transcription and cDNA synthesis were performed as described in a previous study [23] using CFX96-Touch (Bio-rad, Hercules, CA, USA) and SYBR Premix EX Taq™ (Takara, Otsu, Japan). The mouse BDNF, CREB, activity-regulated cytoskeleton-associated protein (ARC), B-cell lymphoma-X1 (BCL-X1) and 18S RNA primers used for the PCR were as follows-for BDNF: sense, 5′-TTG TTT TGT GCC GTT TAC CA-3′, anti-sense, 5′-GGT AAG AGA GCC AGC CAC TG-3′; for CREB: sense, 5′-AAT GGT ACG ATG GGG TAC A-3′, anti-sense, 5′-TCC ATC AGT GGT CTG TGC AT-3′; for ARC: sense, 5′-GAG AGC TGA AAG GGT TGC AC-3′, anti-sense, 5′-GCC TTG ATG GAC TTC TTC CA-3′; for: BCL-X1: sense, 5′-TTC GGG ATG GAG TAA ACT GG-3′, anti-sense, 5′-TGT CTG GTC ACT TCC GAC TG-3′; and for 18S RNA: sense, 5′-TAA CCC GTT GAA CCC CAT T-3′, and anti-sense, 5′-CCA TCC AAT CGG TAG TAG CG-3′. We amplified cDNA using the following conditions: 95 °C for 2 min, followed by 40 cycles for 15 s at 95 °C, and 35 s at 60 °C. All results were standardized to 18S RNA gene expression, and relative mRNA transcript levels were determined by the Ct formula. Each sample was run in triplicate, and the average of the three measurements was calculated for every sample.

### 2.6. Western Blot

Hippocampus samples obtained at the end of the animal experiment were homogenized in lysis buffer containing 1% protease inhibitors. Approximately 15 µg proteins were moved to a new tube and incubated at 100 °C for 5 min for denaturation. Sodium dodecyl sulfate-polyacrylamide gel electrophoresis was run at 120 V for 45 min. The proteins were transferred to polyvinylidene difluoride membranes and then blocked with 5% non-fat dry milk buffer for 60 min at room temperature (RT). Blots were incubated with anti-phospho-p44/42 mitogen-activated protein kinase (MAPK), anti-p44/42 MAPK (ERK1/2), anti-CREB, anti-phospho-CREB, anti-AKT (Cell Signaling Technology, Boston, MA, USA), and anti-phospho-AKT (Abcam, Hong Kong, China) antibodies overnight at 4 °C. After washing three times, the membranes were incubated with secondary antibody for 45 min at RT. ^e^ECL Western Blot Kit (Beijing CoWin Biotechnology, Beijing, China) was used to develop the bands.

### 2.7. Statistical Analysis

All experiments were independently performed twice, and each experiment was conducted using five or ten samples. Data are presented as mean ± SEM. Significant differences between groups were determined by one-way ANOVA, followed by two-tailed multiple *t*-tests using the Student–Newman–Keuls method in SPSS biostatistics software (IBM, Armonk, NY, USA). Statistical significance was considered at *p* < 0.05.

## 3. Results

### 3.1. NGF-Mimicking Effects of PSE, FO, and MPF on PC12 Cells

**Figure 1:**
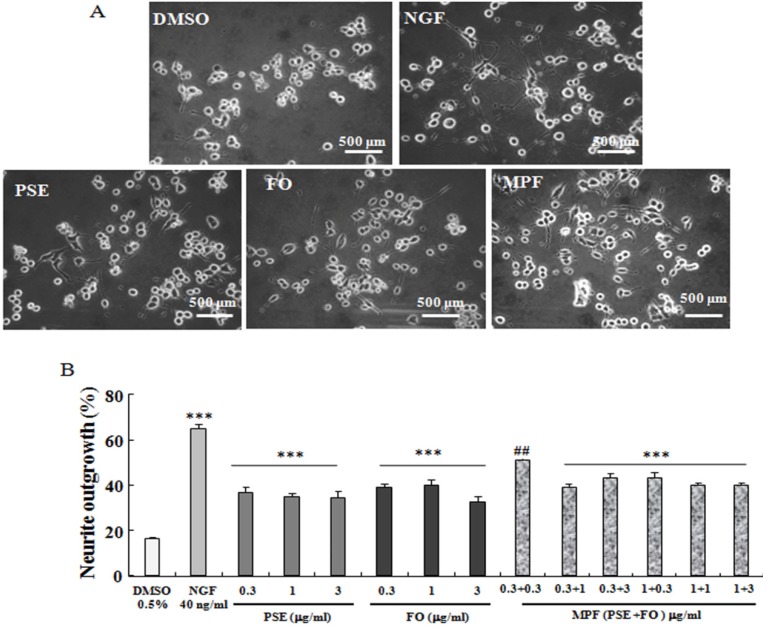
NGF-mimicking effects of PSE, FO and MPF on PC12 cells. (**A**) microphotograph of PC12 cells after treatment with DMSO, NGF, PSE, FO and MPF for 48 h; (**B**) percentage of PC12 cells with neurite outgrowth after treatment with DMSO, NGF, PSE, FO and MPF for 48 h. Cells bearing neurites were identified as those with processes that were at least twice the cell diameter in length. (Control: DMSO, 0.5%; Positive control: NGF, 40 ng/mL). *** Significantly different from the control group at the same time point at *p* < 0.001. ^##^ Significantly different compared to only PSE or FO treated group at a dose of 0.3 μg/mL.

The NGF-mimicking effects of PSE, FO, and MPF on PC12 cells are displayed in Figure 1A–B. PSE-induced neurite outgrowth in PC12 cells, and the percentages of neurite-bearing cells for those treated with 0.3, 1.0, and 3.0 µg/mL PSE for 48 h reached 37.0% ± 2.4%, 34.7% ± 1.3%, and 34.0% ± 3.3%, respectively. These values were significantly higher than that of the control group (16.0% ± 2.4%, *p* < 0.001). FO induced neurite outgrowth, and the percentages of neurite-bearing cells for those treated with 0.3, 1.0 and 3.0 µg/mL FO for 48 h reached 39.0% ± 1.7%, 43.3% ± 2.4%, and 32.5% ± 2.0%, respectively (*p* < 0.001). The percentages of neurite outgrowth after treatment with 0.3 + 0.3, 0.3 + 1.0, 0.3 + 3.0, 1.0 + 0.3, 1.0 + 1.0, and 1.0 + 3.0 µg/mL PSE + FO (MPF) for 48 h reached 50.7% ± 0.6%, 37.0% ± 1.2%, 43.0% ± 2.4%, 43.0% ± 2.5, 39.5% ± 1.2%, and 40.0% ± 1.3%, respectively (*p* < 0.01, *p* < 0.001). These results suggest that PSE, FO and MPF significantly influence neurite outgrowth of PC12 cells.

### 3.2. MPF Improves Learning Ability and Spatial Memory

**Figure 2:**
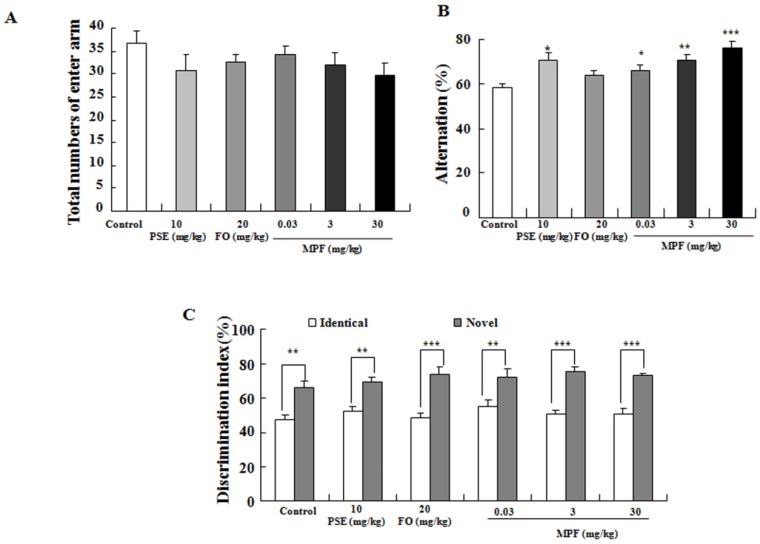
Effects of PSE, FO, and MPF on the learning and memory of mice *in vivo*. (**A**) changes in total numbers of enter arm; (**B**) alternation in the Y-maze test after treating PSE, FO and MPF; (**C**) change in cognitive index in the novel object recognition test after treating PSE, FO and MPF. Y maze and NOR tests were performed after sample administration for five weeks. Each value represents the mean ± SEM of eight or seven mice. *^,^ ** and *** indicate significant difference relative to the control group at the same time point at *p* < 0.05, *p* < 0.01 and *p* < 0.001, respectively.

Examination of animal behavior is a highly important part for functional evaluation. The Y-maze and NOR tests were performed to evaluate the behavior of PSE, FO, MPF treated mice and control mice. The total numbers of enter arms did not change in any of the groups (Figure 2A). Hence, the spontaneous alternation in PSE group at 10 mg/kg (70% ± 3.3%) and MPF treated group at 3 (71% ± 2.6%) and 30 mg/kg (76% ± 3.3%) were obviously higher than that of the control group (58% ± 2.1%, *p* < 0.05, *p* < 0.01 or *p* < 0.001) (Figure 2B). During the NOR test, all animals in the training phase showed similar DIs for recognition of the two familiar objects (Figure 2C). In the trial phase, all treated mice receiving PSE, FO and MPF spent more time exploring the novel object (69% ± 2.7%, 73% ± 4.8%, 72% ± 4.1%, 76% ± 3.2% and 73% ± 2%) than the control mice (66% ± 4%). These results reveal that PSE, FO and MPF enhance the learning ability of mice *in vivo*.

**Figure 3:**
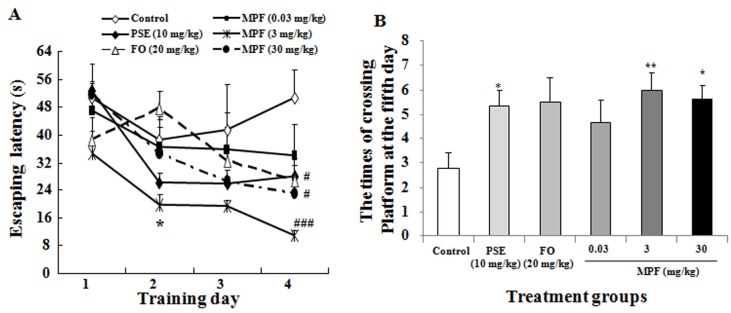
Effects of PSE, FO, and MPF on the spatial memory of mice *in vivo*. (**A**) Escape latency of mice during the training period in water maze test after administrating PSE, FO and MPF; (**B**) Times of crossing platform after training for 4 days in water maze test after administering PSE, FO and MPF. Water maze test was conducted after sample administration for 5 weeks. Each value represents the mean ± SEM of eight or seven mice. ^#^ and ^###^ indicate significant difference relative to the control at the same time point at *p* < 0.05 and *p* < 0.001; * and ** indicate significant difference relative to the control at the same time point at *p* < 0.05 and *p* < 0.01, respectively.

To further evaluate spatial memory, the water maze test was used to assess the training over four days using the hidden platform. A probe trial was conducted without a platform on the fifth day. PSE, FO and MPF did not significantly affect the latency time before training for two days. The latency time significantly reduced in the MPF groups at doses of 3 mg/kg on the second (19.88 ± 2.71) and fourth training day (10.86 ± 1.64) compared with that in the control group at the same time point (38.59 ± 6.84, 50.5 ± 8.3, *p* < 0.05, *p* < 0.001) (Figure 3A). At the same time, the significant reduction of latency time in PSE group was also observed on the fourth training day (28.09 ± 3.10, *p* < 0.05). The crossing platform times in the PSE at 10 mg/kg and MPF-treated groups at 3 and 30 mg/kg (5.3 ± 0.7, 6.0 ± 0.7 and 5.5 ± 0.8) also significantly increased on the fifth day in the probe trial *versus* the control (2.8 ± 0.8, *p* < 0.05 and *p* < 0.01) (Figure 3B). These results indicate that MPF can improve the learning ability and spatial memory of normal mice.

### 3.3. MPF Can Rescue SOD Activity Reduction and Reduce MDA Production Induced by FO in the Plasma

**Figure 4:**
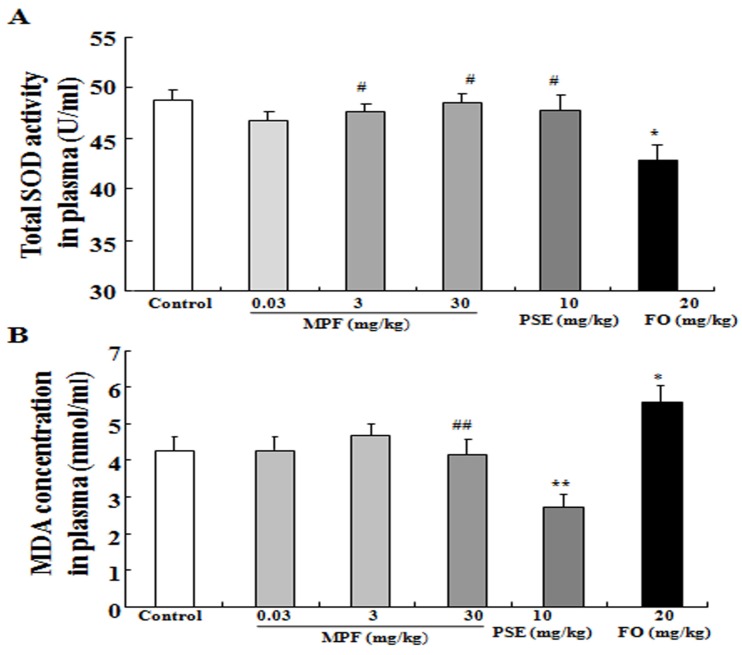
Effects of PSE, FO, and MPF on the SOD activity and MDA level of the plasma. Effects of PSE, FO, and MPF on the SOD activity (**A**) and MDA level (**B**) of the plasma after oral administration of PSE, FO, and MPF for six weeks. Each value represents the mean ± SEM of eight or seven mice. *^,^ ** indicates significant difference relative to the control group at the same time point at *p* < 0.05 and *p* < 0.01; ^#^ and ^##^ indicates significant difference relative to the FO group at the same time point at *p* < 0.05 and *p* < 0.01, respectively.

The changes in SOD activity and MDA level in the plasma after administering MPF, PSE, and FO are displayed in Figure 4A,B. The total SOD activity of the FO group (42.8 ± 1.62) was lower than that of the control group (48.8 ± 1.0, *p* < 0.05). However, the reduction in SOD activity in the FO group can be alleviated by adding PSE to FO (MPF) (47.7 ± 0.8, 48.6 ± 0.9; *p* < 0.05, *p* < 0.05, respectively). The MDA level increased in the plasma of the FO group (5.6 ± 0.5) and reduced in the plasma of the PSE group (2.7 ± 0.3), respectively, compared with that in the control group (4.2 ± 0.1, *p* < 0.05, *p* < 0.05, respectively). The plasma MDA level in the MPF groups at 3 and 30 mg/kg (4.8 ± 0.3, 4.1 ± 0.4) was normalized. These results suggest that using a single FO can induce oxidative stress *in vivo* and that PSE can inhibit the oxidative stress caused by FO.

### 3.4. MPF Increases SOD Activity and Lowers MDA Production in the Cerebral Cortex and Hippocampus

**Figure 5:**
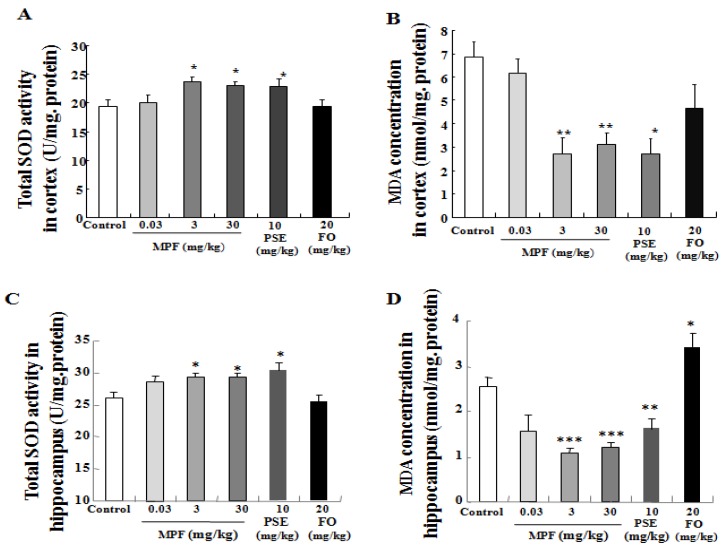
Effects of PSE, FO, and MPF on the SOD activity and MDA level of the cerebral cortex and hippocampus. Effects of PSE, FO, and MPF on the SOD activity and MDA level of the cerebral cortex (**A**, **B**) and hippocampus (**C**, **D**) after oral treatment for six weeks. Each value represents the mean ± SEM of eight or seven mice. *^,^ ** and *** indicate significant difference relative to the control at the same time point at *p* < 0.05, *p* < 0.01 and *p* < 0.001, respectively.

Oxidative stress in the brain impairs the memory and learning ability of mice. Therefore, we also investigated changes in SOD activity and MDA level in the cerebral cortex and hippocampus after treatment. In the cortex, no difference in SOD activity was observed in the FO-treated group (19.3 ± 1.2). However, SOD activity increased in the PSE (23.0 ± 1.2) and MPF groups at 3 and 30 mg/kg (23.8 ± 0.7, 23 ± 0.6) compared with that in the control group (19.4 ± 1.1) (Figure 5A, *p* < 0.05, *p* < 0.05, *p* < 0.05, respectively). The MDA level was significantly lower in the PSE and MPF groups (2.7 ± 0.6, 2.7 ± 0.5, 3.4 ± 0.7) compared with that in the control group (6.8 ± 0.7) (Figure 5B, *p* < 0.05, *p* < 0.01 and *p* < 0.01, respectively). The MDA level in the FO group (4.6 ± 1.0) was decreased but showed no statistically significant differences. In the hippocampus, SOD activity was also significantly increased in PSE (30.7 ± 1.17) and MPF groups at 3 and 30 mg/kg (29.26 ± 0.79, 29.57 ± 0.58) compared with that in the control group (26.1 ± 0.81) (Figure 5C, *p* < 0.05, *p* < 0.05 and *p* < 0.05). The MDA level of the FO group (3.44 ± 0.29) was significantly increased (Figure 5D, *p* < 0.05). Meanwhile, the MDA levels in the PSE and MPF groups at 3 and 30 mg/kg (1.65 ± 0.18, 1.08 ± 0.1, 1.12 ± 0.06) were significantly lowered compared with that of the control group (2.56 ± 0.20) (Figure 5D, *p* < 0.01, *p* < 0.001 and *p* < 0.001, respectively). These results indicate that MPF improves the memory and learning ability of mice by reducing oxidative stress.

### 3.5. MPF Increases BDNF and CREB Gene Expression Levels in the Cerebral Cortex and the Hippocampus

**Figure 6:**
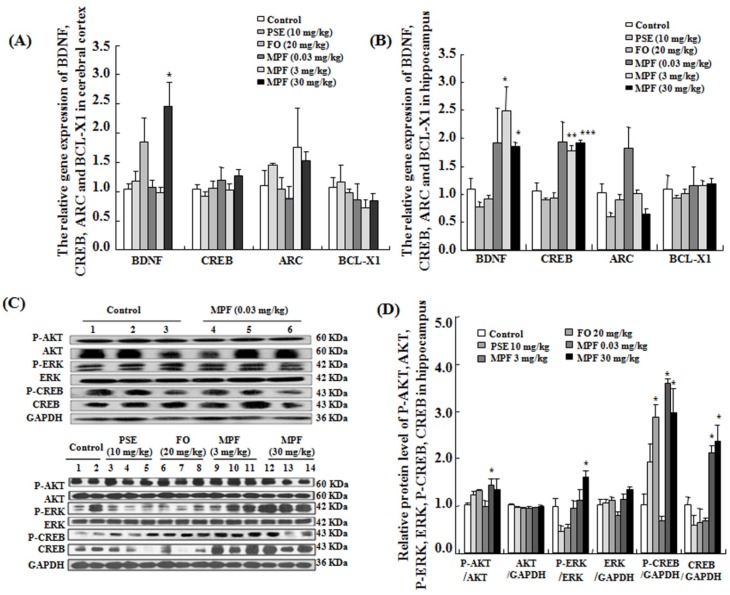
Effects of PSE, FO, and MPF on gene expression, and Western blot analysis in the cerebral cortex and hippocampus. Effects of PSE, FO, and MPF on expression of CREB, BDNF, ARC, and BCL-X1 in the cerebral cortex (**A**) and the hippocampus (**B**), and phosphorylation of AKT, ERK, and CREB proteins in the hippocampus (**C** and **D**). Each value represents mean ± SEM of seven mice. *, ** and *** indicate significant difference relative to the control at the same time point at *p* < 0.05, *p* < 0.01 and *p* < 0.001, respectively.

The gene expression levels of BDNF, CREB, ARC, and BCL-X1 in the cerebral cortex are displayed in Figure 6A. MPF did not affect the mRNA expression levels of CREB, ARC, and BCL-X1 in the cerebral cortex. Only BDNF gene expression was significantly increased in the cerebral cortex (*p* < 0.05). However, changes in both BDNF and CREB gene expression levels were observed in the hippocampus (Figure 6B, *p* < 0.05, *p* < 0.01 and *p* < 0.001). These results suggest that MPF can promote the expression of genes related to memory and learning ability in the hippocampus.

### 3.6. MPF Increases Phosphorylation of AKT, ERK and CREB in the Hippocampus

The hippocampus is critically important for memory and learning. It receives sensory information from the five senses and adjusts endocrine activity. Therefore, we investigated phosphorylation of AKT, ERK, and CREB proteins related to memory and learning in the hippocampus. Phosphorylation of AKT, ERK and CREB was found to be significantly increased in the MPF groups (Figure 6C,D, *p* < 0.05, *p* < 0.05 and *p* < 0.05, respectively). These results suggest that MPF may improve the memory and learning ability of mice via the ERK signaling pathway.

## 4. Discussion

This report demonstrated that PSE, FO and MFP had NGF-mimicking effects on PC12 cells (Figure 1A,B). It is consistent with our previous study [20]. Furthermore, we found that MPF and PSE can enhance both learning and special memory of mice (Figure 2 and Figure 3). However, FO alone can improve the learning ability but not the spatial memory of normal mice (Figure 2 and Figure 3). This result agreed with other reports [24].

In this study, the effects of MPF did not display a dose-dependent relationship; it is possible that normal mice were used to do experiments since we mainly considered the health functions of MPF for healthy people. The index of normal mice was difficult to change by a large margin. Thus, we will investigate the treatment effects of MPF with a pathological model in the future.

Oxidative stress has been implicated in the pathogenesis of dementia and neurodegenerative disorders. Moreover, increases in reactive oxygen species have been implicated in cognitive decline of the aging brain and in AD [25,26,27]. SOD and MDA are closely related to the redox state of an organism. MDA is a lipid peroxide product that indicates aging. Therefore, we focus on these two points to investigate the mechanisms of action of FO, PSE, and MPF in this study. Administration of FO alone for six weeks significantly reduced SOD activity and increased MDA level in the plasma (Figure 4). These results agree with previous reports [28]. As expected, MPF alleviated this symptom and normalized the MDA level. These results indicate that FO induces oxidative stress after long-term administration *in vivo* and that adding PSE can alleviate the side effects of FO. The observed an increase in SOD activity and reduction of MDA production in the cerebral cortex and hippocampus (Figure 5), after treating the samples suggested that long-term administration of FO alone cannot confer neuroprotection *in vivo* and that PSE can alleviate the side effects of FO. MPF and PSE can also significantly improve the memory of mice via anti-oxidative effects.

Memory is known to be regulated by key genes such as BDNF, CREB and ARC. BDNF and CREB participate in memory formation and storage [29,30,31,32]. ARC is an immediate early gene that mediates consolidation of long-term potentiation by altering actin dynamics [30]. Increasing BDNF, CREB, and ARC levels enhance some forms of long-lasting memory. Our observation of elevated expression of BDNF and CREB in the hippocampus (Figure 6B) suggests that BDNF/CREB signaling pathways may have important roles in the enhanced intelligence of MPF-treated mice. We used normal mice in this study, and sensitivity to MPF in the hippocampus was higher than that in the cerebral cortex. PI3K/AKT-signaling pathway is also important for spatial and working memory and amygdala-dependent fear conditioning, whereas the ERK1/2 cascade is involved in an aversively motivated hippocampus and amygdala-dependent learning tasks [33,34,35]. Thus, we investigated the phosphorylation of AKT, ERK, and CREB proteins in the hippocampus using Western blot analysis. The significant increase in the phosphorylation of AKT, ERK and CREB (Figure 6C) indicates that the ERK signaling pathway is also involved in improving the memory of MPF-treated mice.

## 5. Conclusions

In conclusion, FO and PSE exhibited NGF-mimicking effects on PC12 cells and improved the learning ability of normal mice. PSE alleviated the side effects of FO produced in the body after long-term administration, and MPF significantly improved the learning ability and spatial memory of mice via modulation of anti-oxidative stress and regulation of BDNF/ERK/CREB signaling pathways.

## Abbreviations

The following abbreviations are used in this article:

AD	Alzheimer’s disease
AKT	Protein kinase B
ARC	Activity-regulated cytoskeleton-associated protein
BCL-X1	B-cell lymphoma-X1
BDNF	Brain derived-neurophic factor
CREB	cAMP responsive element-binding protein
DHA	Docosahexaenoic acid
DI	Discrimination index
DMSO	Dimethyl sulfoxide
EPA	Eicosapentaenoic acid
ERK	Extracellular signal-regulated kinase
FO	Fish oil
ICR	Institute of Cancer Research
MAPK	Mitogen-activated protein kinase
MDA	Malonyldialdehyde
MPF	Mixture of peanut skin extract and fish oil
NGF	Nerve growth factor
NOR	Novel object recognition
PC12 cells	Rat pheochromocytoma cells
PSE	Peanut skin extract
RT	Room temperature
SOD	Superoxide dismutase
